# Third molar in the orbital floor: A precarious presentation of odontogenic keratocyst—A case report and review of literature

**DOI:** 10.1002/ccr3.1714

**Published:** 2018-08-08

**Authors:** Premalatha Shetty, Sameep Shetty, Nancy Agarwal, Pritika Srivastava

**Affiliations:** ^1^ Department of Oral and Maxillofacial Surgery Manipal Academy of Higher Education Manipal College of Dental Sciences Mangalore India; ^2^ Department of Oral and Maxillofacial Surgery Manipal College of Dental Sciences Mangalore India

**Keywords:** ectopic, odontogenic keratocyst, orbital floor, third molar

## Abstract

The case illuminates the likelihood of a dental pathology presenting with discrete signs and symptoms and the importance of the differential diagnosis of some incongruent clinical entities. The purpose of this article was to present a case report of odontogenic keratocyst (OKC) arising in the orbital floor, finding the common thread in rare pathology, and highlighting the aberration in our treatment plan by collating all the wealth of information published in the literature.

## 1 INTRODUCTION

We describe a case of a 22‐year‐old woman with a vague pain on the left side of the face, episodic headaches, diplopia, and intermittent epiphora, which following a clinical examination and investigations were attributed to an ectopic third molar in the floor of the orbit. Physicians from other streams of medicine were unable to identify the source of pain. The ectopic tooth caused by odontogenic keratocyst (OKC) was enucleated with complete resolution of symptoms. We aim to capture the essence of current knowledge pertaining to OKC and discuss the location and recurrence rate with the treatment options available.

Tooth development results from a complex array of interactions between the oral epithelium and the underlying mesenchymal tissue. A disruption in the tissue interactions during tooth development may potentially result in ectopic tooth development and eruption.^1^

Ectopic placement of third molars is relatively rare. Few anatomic sites where they can reside include the mandibular ramus, coronoid process, maxillary sinus, osteomeatal complex, sigmoid notch, and pterygomandibular space.^2^ Ectopic third molar can be left in situ and safely observed but once associated with a cyst or tumor can invade the dentoalveolar apparatus and shed its cloak of invisibility with few bizarre symptoms that can perplex the clinician.

Trauma, atypical eruption, irregular development of the tooth germ, and odontogenic pathologies are all theories that have been hypothesized. A handful of cases have been reported of tooth in the orbital floor caused by dentigerous cyst that expands the cortical plates may involve other teeth and cause destruction of tissues.^3^ In contrast, OKC tends to grow in an anteroposterior direction within the medullary cavity of the bone without causing obvious bone expansion.^4^ Displacement of teeth adjacent to the OKC occurs more frequently than resorption^5^; however, tooth pushed to the orbital floor due to OKC has been reported sparsely. Based on 2017 classification, keratocystic odontogenic tumor (KCOT) has been subsumed, as OKC. The consensus panel acknowledged that there was some evidence in 2005 to reclassify the cyst to tumor. But currently, the evidence is not sufficient to justify the reclassification. The evidence for reclassification was based on aggressive growth, recurrence after treatment, rare occurrence of a solid variant of OKC, and mutations in the PTCH gene. Apparently, PTCH gene mutations have been reported in up to 85% of nonsyndromic and 30% of syndromic OKC.

The consensus opined the unremitting nature of a pathology to be categorized as neoplasm, which does not hold good in case of OKC. There is substantial evidence of complete regression of OKC following decompression and the lining of many decompressed cysts transform to normal oral mucosa than OKC histologically. Hence, the evidence to classify the cyst as neoplasm is currently lacking to justify the continuation of KCOT.^6^

The aim of this clinical report was to highlight the need for diligent clinical examination aided by mandatory investigations before we dismiss the source of pain as nonodontogenic.

## 2 CASE REPORT

**Figure 1:**
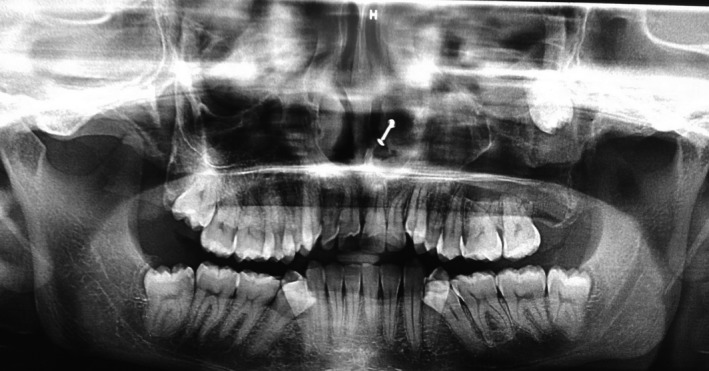
Orthopantomogram showing the presence of an ectopic tooth in the orbital floor

**Figure 2:**
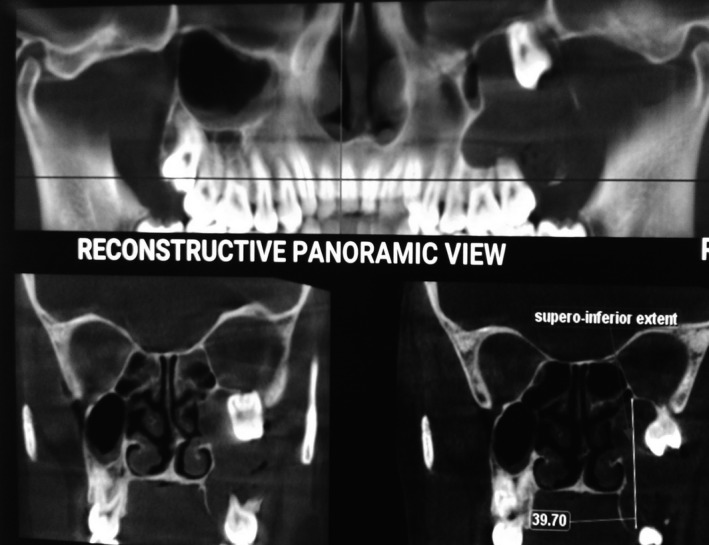
3D reconstructed image showing the presence of an ectopic tooth inside the orbital floor

**Figure 3:**
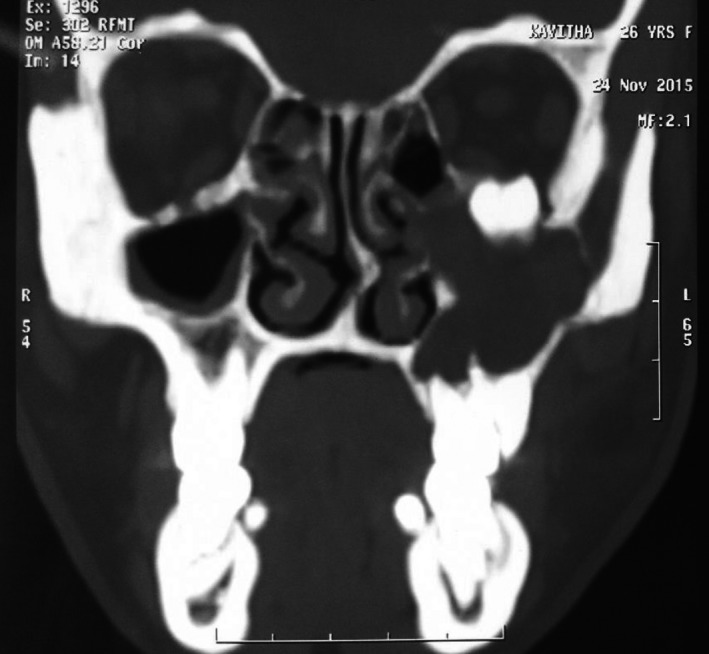
3D reconstructed image showing the presence of an ectopic tooth inside the orbital floor

**Figure 4:**
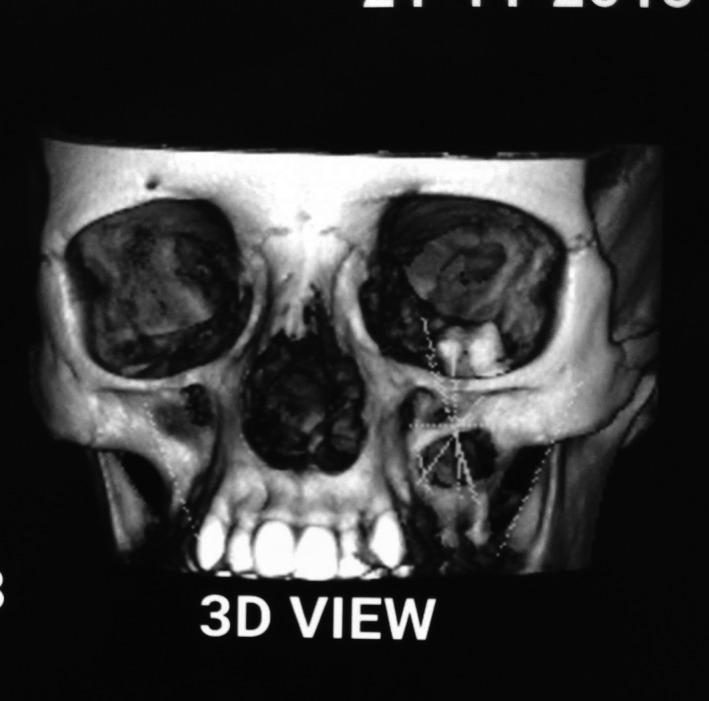
3D reconstructed image showing the presence of an ectopic tooth inside the orbital floor

**Figure 5:**
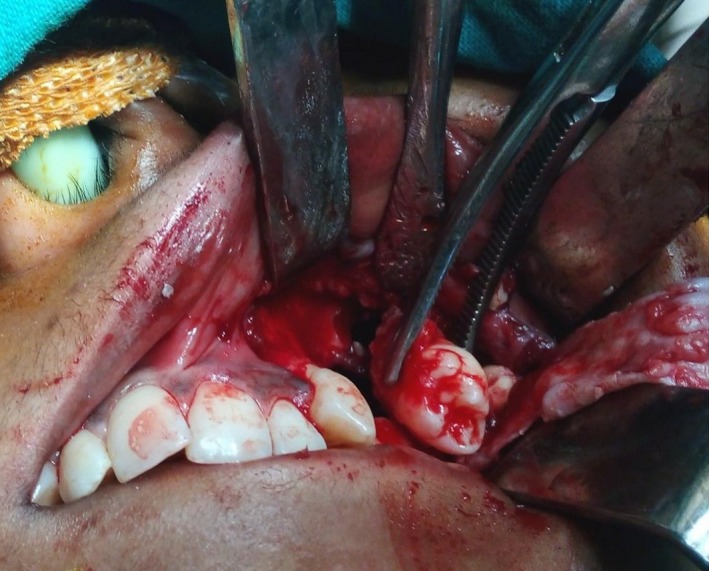
Caldwell approach was performed to retrieve the tooth and enucleate the tumor

**Figure 6:**
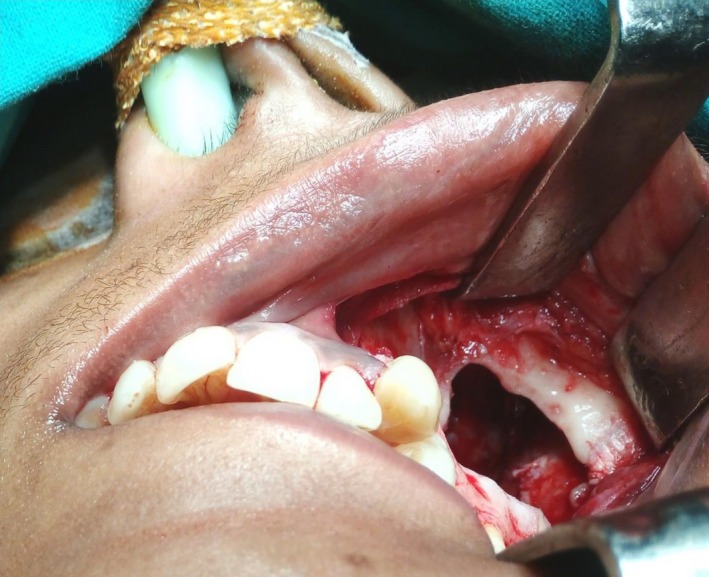
Caldwell approach was performed to retrieve the tooth and enucleate the tumor

**Figure 7:**
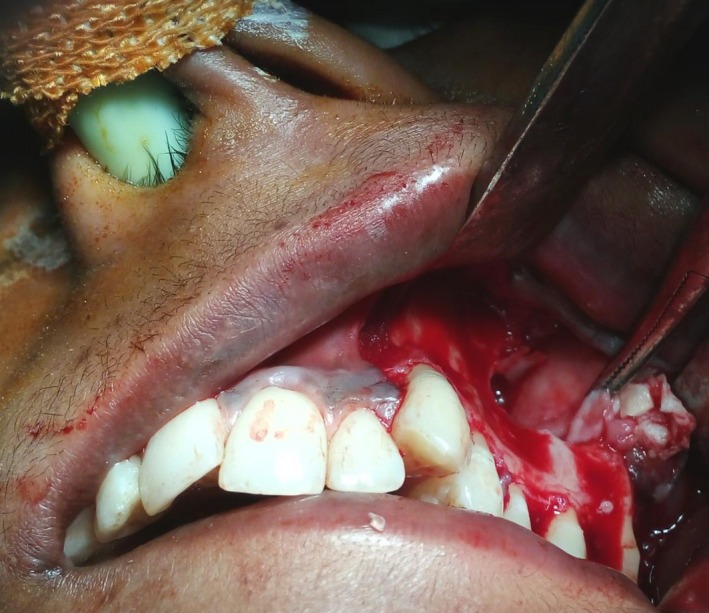
Trapezoidal flap raised to gain access to the tumor

**Figure 8:**
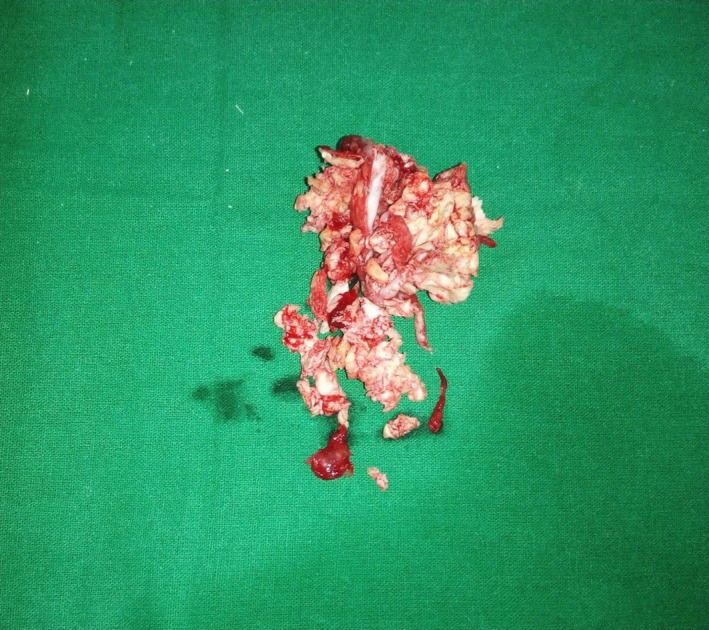
Parakeratinized variant of keratocystic odontogenic tumor (KCOT)

**Figure 9:**
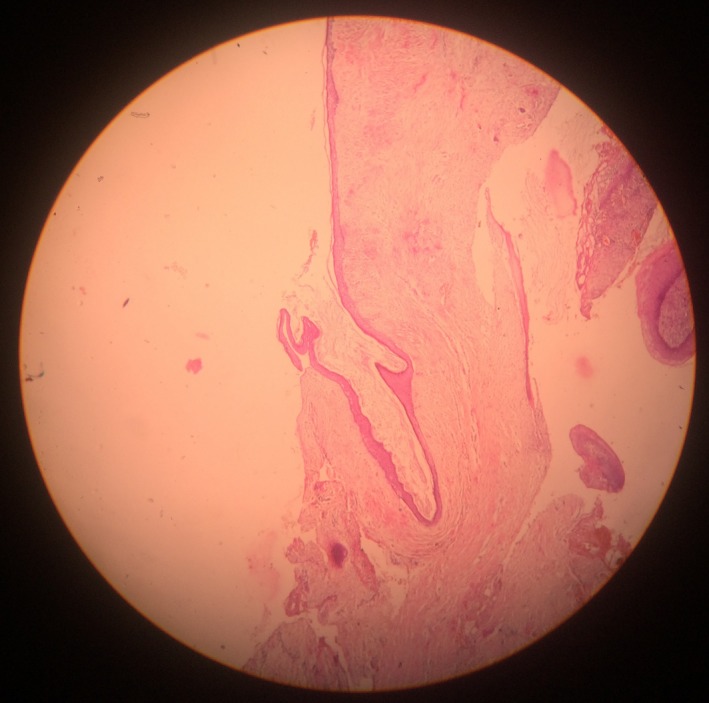
Low power H&E‐stained histologic slide of a typical odontogenic keratocyst (OKC) made up of thin and uniform keratinized stratified squamous epithelium separated from the underlying connective tissue wall (magnification ×200)

**Figure 10:**
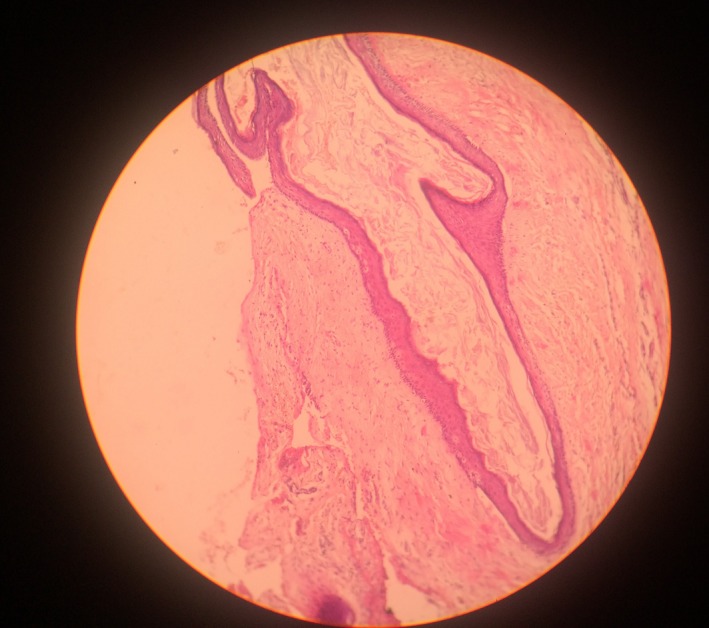
Low power H&E‐stained histologic slide of a typical odontogenic keratocyst (OKC) made up of thin and uniform keratinized stratified squamous epithelium separated from the underlying connective tissue wall (magnification ×200)

A 22‐year‐old female patient reported to us with a vague pain on the left side of face which was throbbing in nature and radiating to the eye on the same side since 18 months. She was apparently in a good health and her medical history was unremarkable. She reported a transient diplopia in an upward gaze and epiphora occasionally. The epiphora could be ascribed to the tumor expansion causing compression of the nasolacrimal duct.^7^ On examination, there was vestibular obliteration extending from the left first premolar up to the second molar. Crepitations were palpated over the premolar region signifying a cystic lesion. Aspiration yielded cheesy material consisting of keratin flakes. Surprisingly, no symptoms of sinusitis were present. Orthopantomogram revealed a high posterior impacted third molar in the maxilla abutting the floor of the orbit. (Figure 1) Further radiological investigations included a CT scan. (Figures 2, 3 and 4) The Caldwell‐Luc operation involves creating an opening into the maxillary antrum through the canine fossa. (Figures 5 and 6) A trapezoidal flap was raised to gain access to the underlying pathology. (Figure 7) Fenestration on the bony wall exposed the keratin cheesy lining which was enucleated along with peripheral ostectomy that aids in the removal of any traces of epithelial remnants. The intrinsic bony erosion enabled us to trace and remove the third molar. The antrum was copiously irrigated with an antral pack in place, removed subsequently. A middle meatus nasal antrostomy was performed to attain a physiological sinus drainage. To assure total removal of the cyst lining, an endoscopy was performed along with a clinical and radiological follow‐up on a regular basis. Histopathology of the excised specimen read as parakeratinized variant of OKC. (Figures 8, 9 and 10) The wound healing was uneventful, with remission of epiphora, and diplopia following a 1‐year follow‐up.

## 3 DISCUSSION

The term OKC, first introduced by Philipsen (1956), reflects its histogenesis as well as the characteristics of its keratinized lining epithelium, which is clearly discernible from other odontogenic cyst types. Its high recurrence rate and association with the nevoid basal cell carcinoma syndrome (NBCCS) seat OKC up in the pyramid of aggressive odontogenic lesions.^8^

Odontogenic keratocyst is a cystic lesion of odontogenic origin that demonstrates the behavioral characteristics of a benign neoplasm and has a propensity to recur after surgical treatment.^9^ Microscopically, it has a uniform epithelial lining of four to eight cells in thickness. The epithelial surface characteristically consists of either parakeratin or orthokeratin, but share a similar clinical presentation.^10^ It has long been acknowledged that in addition to OKC, the majority of odontogenic cysts produce orthokeratin. Orthokeratinized odontogenic cyst (OOC), described by Schultz in 1927, was considered to be a variant of OKC at the outset.^11^

Odontogenic keratocysts have distinctive histologic feature that can distinguish them from other cysts. It is characterized by a uniform epithelial layer that lacks rete ridges. In addition, it has a corrugated parakeratinized luminal layer and a prominent basal cell layer. OKCs have a high recurrence rate and develop more aggressively than any other jaw cysts.

Odontogenic keratocyst may develop at any age, but presents most commonly in the second or third decade of life, usually in males. Multiple OKC may be associated with Gorlin's syndrome (basal cell nevus syndrome). An OKC usually presents as an asymptomatic or painful mass of the posterior mandible or maxilla. Alternatively, it may be discovered on routine radiographic examination as a unilocular or multilocular lytic lesion with well‐defined, scalloped, and sclerotic borders. The average diameter of an OKC is 20 mm with a range of 5‐70 mm. It may displace the roots of teeth, extend into the maxillary antrum, or result in pathologic fractures of the mandible.^10^

The incidence of OKC fortunately in the maxilla is very less compared to the mandible. (Table 1 shows the locations of OKC in the head and neck regions by reviewing 31 articles.) The region of OKC in the maxilla can be fatal due to the following reasons^12^:

**Table 1:** Locations of OKC in the various regions of maxilla and mandible

Authors	Anterior mandible	Posterior mandible	Anterior maxilla	Posterior maxilla	Both	Maxillary sinus and Ethmoidal sinus	Cavernous sinus	Orbit	Palate
Joseph^24^					1				
Hugh^10^							1 (left)	1 (post)	
Byakodi^25^		1							
Byatnal^26^		1							
Macdonald^27^		4		1					
Bordello^27^	4								
Wright^27^	4	30	10	10					
Chiang^27^		1	1						
Siar^27^	5	3	1						
Vuhahula^27^	8		4						
Li (10 y)^27^		6							
Li (17 y)^27^	1	7		1					
Santos^27^	3		5						
Li (2003)^27^	18		2						
Grossman^27^	3		5						
Dong^28^	68	276	57	60					
Bhasin^29^									1
Sarvaiya^30^		1							
Jones^31^		236							
Kaczmarzyk^15^		81		27					
Swain^32^	1								
Mahdavi^33^			1						
Galvan^34^		3							
Sarkar^35^	1								
Bharathi^36^	1								
Rensburg^37^	4	16	1	7		1			
Pogrel^38^		1							
Stoelinga^39^		1							
Shear^40^		94		31					
Chuong^41^								1	

The thin cortical bone can be easily perforated to gain access to distant locations.

Hollow cavities: maxillary sinus and nasal cavity: a fully permeable barrier for tumors to ingress.

Camouflaging radiographic features of the OKC and maxillary sinus, which are often difficult to interpret even with a CT scan.

Perforation into the pterygopalatine spaces rendering the tumor inaccessible and unresectable.

Application of Carnoy's solution a chemical adjunct to decrease the recurrence rate is contraindicated due to the continuity of the tumor with sinus and the orbital floor.

Tooth displacement into the various head and neck spaces can be due to disturbances in the multistep interactions during tooth eruption process (breach in the complex interaction between the oral epithelium and underlying mesenchymal tissue), iatrogenic or can be one of the covert manifestations of aggressive odontogenic cyst and tumors. The other etiological factors that can push the tooth include developmental disorders such as cleft palate, trauma, infection, crowding, genetic factors, and high bone density.^13^

Individuals afflicted with nevoid basal cell carcinoma syndrome are young with multiple lesions which were absent in our case. Recurrences are higher with nevoid basal cell carcinoma syndrome, approximately 50% and with OKC diagnosed in second and eighth decade. Patients may seek care for maxillary lesions at an advanced stage because of its ability to spread to other structures such as the sinus with no remarkable symptoms leading to a higher recurrence rate and requiring more aggressive management.^14^

Myriad treatments are available (Table 2) to enable complete removal of OKC and minimize recurrence; however, the absolute treatment for OKC is still contentious. The overall recurrence rate of OKC ranges from 16% to 30% (23.15%)^15^. The intricate anatomy, confluence of vital structures near the orbit disabling the use of Carnoy's solution prompted us to go for a conservative approach. We have laudably distilled the surgical options considering the young age, unilocular radiographic appearance of the lesion with no scalloped borders, a localized tumor territory, and esthetics. Liquid nitrogen cryotherapy,^16^ peripheral ostectomy, and endoscopy,^17^ as a complementary method, are recommended to identify some cystic residua and/or limit the probability of recurrence. The clinical symptoms far from the territory of the pathology make this case a challenging one to diagnose.

**Table 2:** Various treatment modalities with recurrence rate

Year	Authors	Title	Treatment modality	Recurrence rate (%)
1991	Brondum and Jensen^42^	Recurrence of keratocysts and decompression treatment: a long‐term follow‐up of 44 cases	Decompression and cystectomy	0
2001	Pogrel et al^43^	The use of enucleation and liquid nitrogen cryotherapy in the management of OKCs	Enucleation with cryotherapy	11.5
2002	Zhao et al^44^	Treatment of OKCs: a follow‐up of 255 Chinese patients	Enucleation and curettage	17.79
			Enucleation with Carnoy's solution	6.70
			Marsupialization alone	0
			Resection	0
			Marsupialization and enucleation	0
2003	Bell et al^9^	Treatment options for the recurrent OKC	Enucleation and curettage	62.5
2005	Morgan et al^45^	A retrospective review of treatment of the OKC	Enucleation with Carnoy's solution	50
			Enucleation with peripheral ostectomy	18.18
			Enucleation with peripheral ostectomy and Carnoy's solution	0
			Resection	0
			Enucleation alone	54.55
2010	Zecha et al^46^	Recurrence rate of KCOT after conservative surgical treatment without adjunctive therapies—a 35‐year single institution experience	Enucleation alone	20.69
			Marsupialization alone	40

KCOT, keratocystic odontogenic tumor; OKC, odontogenic keratocyst.

The recurrence rate associated with enucleation with adjunctive therapy such as cryosurgery and decompression (1‐8 percent) is lower than that associated with enucleation alone (17‐56 percent).^18^ Postoperative follow‐up with regular radiographic examination is important with OKCs because of the potential for recurrence. OKCs usually recur within 5 years after surgery,^19^, ^20^ but can recur more than 15 years later.^21^, ^22^ So, for the first 5 years, patients follow up every year and thereafter every 2 years if possible is recommended.^23^ In this case, postoperative follow‐up was carried out for a period of 1 year along with a clinical and radiological follow‐up on a regular basis.

## 4 CONCLUSION

In conclusion, ectopic tooth in the floor of the orbit caused by an OKC is extremely rare. Third molar is the commonest tooth involved; also being the last tooth to erupt in the oral cavity is more likely to be displaced while competing for space. Clinicians need to be cognizant of missing tooth associated with hidden pathologies and its remote location far off from the dentoalveolar apparatus. A diligent clinical and radiographic evaluation, inclusive of few unusual locations, should be undertaken before dismissing teeth as being avulsed or congenitally missing.

## CONFLICT OF INTEREST

None declared.

## AUTHORSHIP

PS, SS, NA, and PS: operated the case, acquired the data, and prepared the manuscript. PS and SS: defined the intellectual content. PS, SS, and NA: involved in literature search and reviewed the manuscript. SS and NA: edited the manuscript.
